# Preventive role of *Sapindus* species in different neurological and metabolic disorders

**DOI:** 10.17179/excli2021-4625

**Published:** 2022-01-31

**Authors:** Sarita Rawat, Gaurav Gupta, Anurag Mishra, Sachchidanand Pathak, Lakshmi Thangavelu, Sachin Kumar Singh, Niraj Kumar Jha, Deepak Kumar, Poonam Negi, Avvaru Praveen Kumar, Dinesh Kumar Chellappan, Kamal Dua

**Affiliations:** 1 Faculty of Pharmacy and Sciences, Amrapali Group of Institutes, Shiksha Nagar, Lamachaur, Haldwani, 263139, Nainital, Uttarakhand, India; 2 School of Pharmacy, Suresh Gyan Vihar University, Jagatpura 302017, Mahal Road, Jaipur, India; 3 Department of Pharmacology, Saveetha Dental College, Saveetha Institute of Medical and Technical Science, Saveetha University, Chennai, India; 4 Nims Institute of Pharmacy, NIMS University, Jaipur, Rajasthan, India; 5 Kashi Institute of Pharmacy, Varanasi, UP, India; 6 Center for Transdisciplinary Research (CFTR), Department of Pharmacology, Saveetha Dental College, Saveetha Institute of Medical and Technical Sciences, Saveetha University, Chennai, India; 7 School of Pharmaceutical Sciences, Lovely Professional University, Phagwara, Punjab, 144411, India; 8 Department of Biotechnology, School of Engineering and Technology (SET), Sharda University, Uttar Pradesh, Greater Noida, India; 9 Department of Pharmaceutical Chemistry, School of Pharmaceutical Sciences, Shoolini University, Solan - 173229, India; 10 Department of Pharmacy, Shoolini University, Solan - 173229, India; 11 Department of Applied Chemistry, School of Applied Natural Science, Adama Science and Technology University, P.O.Box 1888, Adama, Ethiopia; 12 Department of Life Sciences, School of Pharmacy, International Medical University, Kuala Lumpur 57000, Malaysia; 13 Discipline of Pharmacy, Graduate School of Health, University of Technology Sydney, NSW 2007, Sydney, Australia; 14 Faculty of Health, Australian Research Centre in Complementary and Integrative Medicine, University of Technology Sydney, Ultimo NSW 2007, Australia

## ⁯

*Sapindus, *also known as soap nut is rich in saponins. The tree belongs to the family Sapindaceae which has six to twelve closely related species, primarily comprising of shrubs and small trees. Being one of the world's oldest cultivated medicinal plants, *Sapindus* boasts of various therapeutic uses. The use of this valuable tree has been traced back to the period of Ancient India, which is estimated to be around 5000 years ago. It is a deciduous tree with moderate length that grows naturally in the Southern states of India and also in some regions of Northern India. Sugars, fatty acids, trifoliosides, tannins, phenolic acids, steroids, carbohydrates, and triterpenoids are the primary phytoconstituents derived and reported from different parts of the plant (Arul et al., 2004[1]). *Sapindus* is also used in Ayurvedic composition of shampoos and cleansers as an important component. Some tribes in India, use a decoction of the plant's aerial parts for the treatment of diabetes mellitus, as described in traditional reports. Contemporarily, *Sapindus trifoliatus* (ST) has been used for decades to treat colds caused by infection and inflammation, and is also used in combination with standard medicine to treat a variety of malignancies and conditions such as diabetes mellitus (Arulmozhi et al., 2004[2]). Soap nut powder contains potent antimicrobial activity and because of this it is widely used in cosmetic and contraceptive creams. Arthritis, common cold, constipation, nausea, and dental caries are also treated by using powdered seeds of the plant. It is also beneficial for skin disorders like eczema and psoriasis (Arulmozhi et al., 2004[4]). In addition, *Sapindus* species has also been used for thousands of years in traditional medicine to treat excessive salivation, epilepsy, chlorosis, and neuroleptic diseases (Arulmozhi et al., 2005[3]). Current biological and pharmacological updates on *Sapindus trifoliatus* have been reviewed below (Table 1(Tab. 1); References in Table 1: Arul et al., 2004[1]; Arulmozhi et al., 2004[2][4], 2005[3][5]; Bera et al., 2019[6]; Bodhankar et al., 1974[7]; Borad et al., 2001[8]; Chaudhary et al., 2019[9]; Chen et al., 2019[10]; Desai, et al., 1986[11]; Dixit and Gupta, 1982[12]; Gandreddi et al., 2015[13]; Grover et al., 2005[14]; Hu et al., 2018[15]; Kamboj and Dhawan, 1982[16]; Lal et al., 1976[17]; Lu et al., 2016[18]; Polli et al., 2021[19]; Pore et al., 2010[20]; Porsche et al., 2018[21]; Samiksha et al., 2019[22]; Sirisha et al., 2018[23]; Tiwari et al., 2008[24]; Tungmunnithum et al., 2018[25]; Wei et al., 2021[26]).

## Conflict of interest

The authors declare no conflict of interest.

## Figures and Tables

**Table 1:**
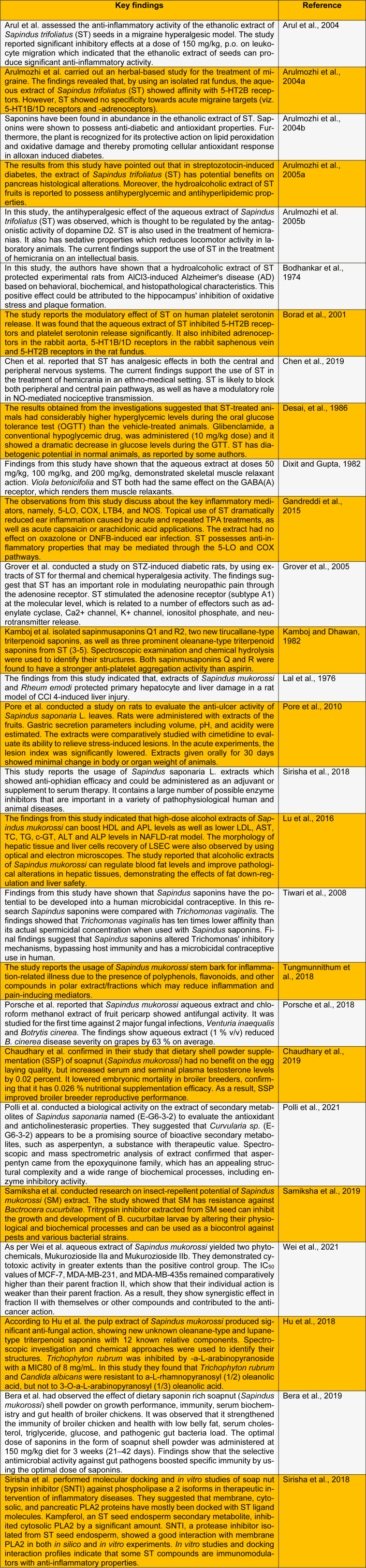
Current biological and pharmacological updates on *Sapindus *species
